# The PLANES study: a protocol for a randomised controlled feasibility study of the placental growth factor (PlGF) blood test-informed care versus standard care alone for women with a small for gestational age fetus at or after 32 + 0 weeks’ gestation

**DOI:** 10.1186/s40814-020-00722-x

**Published:** 2020-11-19

**Authors:** Joanna Gent, Sian Bullough, Jane Harrold, Richard Jackson, Kerry Woolfall, Lazaros Andronis, Louise Kenny, Christine Cornforth, Alexander E. P. Heazell, Emily Benbow, Zarko Alfirevic, Andrew Sharp

**Affiliations:** 1 Harris-Wellbeing Research Centre, University of Liverpool, Liverpool, UK; 2 Liverpool Clinical Trials Unit, University of Liverpool, Liverpool, UK; 3 Department of Public Health, Policy and Systems, Institute of Population Health, University of Liverpool, Liverpool, UK; 4 Division of Health Sciences and Warwick Clinical Trials Unit, University of Warwick, Coventry, UK; 5 Faculty of Health and Life Sciences, University of Liverpool, Liverpool, UK; 6 Maternal and Fetal Research Centre, School of Medical Sciences, University of Manchester, Manchester Academic Health Science Centre, 5th Floor (Research), St Mary’s Hospital, Oxford Road, Manchester, M13 9WL UK

**Keywords:** Fetal growth restriction (FGR), Intrauterine growth restriction, Small for gestational age (SGA), Placenta, Placental growth factor, Soluble fms-like tyrosine kinase

## Abstract

**Background:**

Stillbirth remains a major concern across the globe and in some high-resource countries, such as the UK; efforts to reduce the rate have achieved only modest reductions. One third of stillborn babies are small for gestational age (SGA), and these pregnancies are also at risk of neonatal adverse outcomes and lifelong health problems, especially when delivered preterm. Current UK clinical guidance advocates regular monitoring and early term delivery of the SGA fetus; however, the most appropriate regimen for surveillance of these babies remains unclear and often leads to increased intervention for a large number of these women. This pilot trial will determine the feasibility of a large-scale trial refining the risk of adverse pregnancy outcome in SGA pregnancies using biomarkers of placental function sFlt-1/PlGF, identifying and intervening in only those deemed at highest risk of stillbirth.

**Methods:**

PLANES is a randomised controlled feasibility study of women with an SGA fetus that will be conducted at two tertiary care hospitals in the UK. Once identified on ultrasound, women will be randomised into two groups in a 3:1 ratio in favour of sFlt-1/PlGF ratio led management vs standard care. Women with an SGA fetus and a normal sFlt-1/PlGF ratio will have a repeat ultrasound and sFlt-1/PlGF ratio every 2 weeks with planned birth delayed until 40 weeks. In those women with an SGA fetus and an abnormal sFlt-1/PlGF ratio, we will offer birth from 37 weeks or sooner if there are other concerning features on ultrasound. Women assigned to standard care will have an sFlt-1/PlGF ratio taken, but the results will be concealed from the clinical team, and the woman’s pregnancy will be managed as per the local NHS hospital policy. This integrated mixed method study will also involve a health economic analysis and a perspective work package exploring trial feasibility through interviews and questionnaires with participants, their partners, and clinicians.

**Discussion:**

Our aim is to determine feasibility through the assessment of our ability to recruit and retain participants to the study. Results from this pilot study will inform the design of a future large randomised controlled trial that will be adequately powered for adverse pregnancy outcome. Such a study would provide the evidence needed to guide future management of the SGA fetus.

**Trial registration:**

ISRCTN58254381. Registered on 4 July 2019

## Background

The stillbirth rate within the United Kingdom (UK) remains one of the highest in high-resource countries (3.87 per 1000 births) [1], but stillbirths remain rare at term (2.0 per 1000 births) [2]. Historically strategies to prevent stillbirth have focused on the identification of risk factors in early pregnancy [3–9]. However, risk factors present at booking predict less than 20% of all stillbirths [8]. Furthermore, 33% of stillbirths occur after 36 + 0 weeks, 85% prior to labour with the cause of death unknown in 39% [8]. A third of all stillbirths however are small for gestational age (SGA), and therefore, targeted identification and intervention on the small fetus has become an attractive surrogate strategy to prevent subsequent stillbirth [8]. Even when identified antenatally, SGA fetuses are at a significantly higher risk of stillbirth (odds ratio 7.0, 95% confidence interval 3.3–15.1) [10–12], neonatal adverse outcome [13] and potential life-long health risks [14, 15].

The standard approach advocated by the National Institute for Health and Care Excellence (NICE) for all pregnant women in the UK to identify the SGA fetus relies upon serial measurement of the maternal abdomen with a tape measure from 24 weeks to generate the symphysial fundal height (SFH) [16]. SGA is suspected when the SFH measurement is < 10th centile or there is static growth over two measurements. SFH measurement in isolation has a sensitivity of 30–40% [17, 18] and with no randomised controlled studies of its effectiveness [19]. Therefore, confirmatory ultrasound assessment is required, with SGA commonly defined as an estimated fetal weight (EFW) < 10th centile [4, 17, 20]. However, the increase in detection of SGA with ultrasound is limited, with up to 41% of SGA fetuses remaining undiagnosed and a false positive rate of up to 20% [18].

The Royal College of Obstetricians and Gynaecologists (RCOG) has produced guidance for the management of the SGA fetus [17], but the Cochrane Collaboration acknowledges that the most appropriate regimen for antenatal surveillance is unclear [19]. This guidance advocates that the SGA fetus should have growth assessed with ultrasound every 2 weeks with additional fetal blood flow (Doppler) assessments. Timing of delivery is based upon deterioration in fetal growth or fetoplacental Doppler (< 37 weeks) or when the pregnancy reaches 37 + 0 weeks’ gestation even if all other factors are normal [17]. Therefore, the UK National Health Service (NHS) currently has a system for the management of the SGA fetus and prevention of stillbirth based upon SFH and confirmatory ultrasound with delivery from 37 + 0 weeks. This ‘one size fits all’ approach maintains a safety margin to prevent stillbirth but leads to an increase in interventions, such as induction of labour (IOL) [21]. IOL rates for SGA are increasing (3.0% in 2012 to 10.7% in 2016) with up to 40% of all labours now induced [22].

Our recent survey of UK obstetric units demonstrates that this is a UK-wide phenomenon with mean induction rates at 30% (range 17–46%) [22], with 67% of responders observing an increase over 5 years and 90% stating that in their opinion management of SGA had been a factor. This increased intervention and delivery of SGA fetuses at a late preterm or early term gestation, whilst well intentioned is not without concern. There is a substantial body of evidence showing that being born < 39 weeks has an impact upon a child’s cognitive development and later academic achievement [23–26]. Furthermore, whilst overall numbers of affected children are low, the term SGA fetus has a cerebral palsy risk 5–7 times greater than normal birth weight babies [27, 28]. There is also an impact on women’s choice as to place and mode of birth, especially when in general the risk of stillbirth is low [29] with intervention potentially not required for all SGA fetuses.

### Rationale for study population

The desire to reduce stillbirth is powerful, but due to the relative infrequency of this outcome, it currently leads to increased intervention for a large number of women, which impacts upon a women’s choice and increases the burden on the health care system. However, most importantly whilst small gains have been made in stillbirth reduction in the UK, the goal of significantly reducing stillbirth remains distant. Not all national guidelines are as prescriptive on the management of SGA, recently reviewed by McCowan et al. [30]. Canadian [31] guidance suggests close monitoring of the fetal condition with ultrasound after 37 weeks but with no defined time to deliver. Irish [32], US, and New Zealand guidance [33] is also more flexible suggesting that in the presence of normal Doppler studies the SGA fetus can be left until 38–39 or 40 weeks respectively. Much of this evidence for lack of harm from delaying delivery comes from the DIGITAT study that showed no adverse effects from induction of labour vs delayed delivery [34], though this study was underpowered to offer unequivocal evidence regarding perinatal mortality or severe morbidity. Recently, some reaction against early delivery for SGA in the absence of other risk factors has been observed with a recent UK study deferring delivery until 40 weeks if fetal assessment was normal [35]. However, in this study there was a single stillbirth in the deferred cohort possibly suggesting that additional reassurance of fetal wellbeing is required.

We suggest a potential ‘middle ground’ would be to refine the risk of adverse pregnancy outcome in SGA pregnancies with biomarkers of placental function, namely the sFlt-1/PlGF ratio. We feel that this may help clinicians to differentiate the fetus that is constitutionally small from that which has a reduced growth velocity due to placental failure. Identifying the group at highest risk of stillbirth would reduce the number of interventions performed and reduce the number of babies delivered early whilst maintaining a safety margin to prevent stillbirth. This would represent a more detailed monitoring system than any other nation currently advocates. It would also align with the recent Saving Babies’ Lives Care Bundle, which advocates individualised risk assessments of SGA pregnancies and deferring delivery to 39 weeks in those pregnancies with no high-risk features [36].

### Justification for intervention and biomarkers

The use of biomarkers to identify the fetus at risk of stillbirth has been highlighted as a priority by the RCOG [17] and the James Lind Alliance [37]. However, their low predictive accuracy in the first trimester has limited their use as a screening tool [3]. Recent advances in our understanding of which biomarkers are clinically relevant in late pregnancy has demonstrated that identification of placental disease potentially predisposing to stillbirth is possible [38]. Principal among the biomarkers currently available is placental growth factor (PlGF). This protein is produced by the placenta and identifiable in maternal blood from 12 weeks [39]. Two commercially available platforms which measure PlGF (Alere®) [40] or PlGF relative to sFlt-1 (sFlt-1/PlGF ratio) (Roche®) [41] have been endorsed by NICE for the investigation of hypertension in pregnancy [42]. Whilst the majority of studies have focused on the ability of these tests to predict preeclampsia, there is a significant amount of information on their ability to predict stillbirth and SGA. Abnormally low levels of PlGF in maternal plasma have been linked to preeclampsia [40, 43, 44], SGA [45, 46], and stillbirth [40, 47]. Furthermore, an abnormal PlGF appears to more than double adverse pregnancy outcome [48] and is associated with critical fetal growth restriction [49–53]. In a cohort of SGA fetuses, low PlGF was associated with preterm delivery, stillbirth, birth weight < 3rd centile, Apgar < 7 at 5 min, NICU admission and placental pathology [40, 54, 55]. Women with the lowest PlGF values were in addition much more likely to have a growth-restricted fetus and abnormal Dopplers [40, 47]. In all published studies to date, very few fetuses have been stillborn following a normal PlGF result [40, 47, 53, 56, 57]. The ratio of PlGF to soluble FMS-like tyrosine kinase-1 (sFlt-1), which binds PlGF in the circulation, is increased in preeclampsia [41], fetal growth restriction [58] and stillbirth [59]. An abnormal sFlt-1/PlGF ratio of > 38 is associated with an increased risk of SGA (21% vs 7%) [60]. The sFlt-1/PlGF ratio appears to be equally useful in determining outcome with almost no stillbirths when the ratio is normal [41, 56, 57, 59, 60] (Table 1). A recent Cochrane Diagnostic Test Accuracy Review on the effectiveness of biomarkers to predict stillbirth [61] confirms that abnormal PlGF or sFlt-1/PlGF ratio has a diagnostic odds ratio of 49.2 for subsequent stillbirth. Therefore, PlGF or sFlt-1/PlGF ratio appears to be effective in identifying the fetus that is SGA and more importantly, those fetuses that go on to be stillborn.

**Table 1:** Stillbirths by normal and abnormal PlGF and sFlt-1/PlGF ratio

Author	Year	Stillbirth normal PlGF	Stillbirth abnormal PlGF	Stillbirth normal sFlt-1/PlGF ratio	Stillbirth abnormal sFlt-1/PlGF ratio
**Chappell et al.** [40]	**2013**	0	7	-	-
**Benton et al.** [53]	**2016**	1	6	-	-
**Zeisler et al.** [41]	**2016**	-	-	1	3
**Sovio et al.** [59]	**2017**	-	-	0	0
**Sharp et al.** [47]	**2018**	0	1	-	-
**Sharp et al.** [56]	**2018**	0	35	0	35
**Navaratnam et al.** [57]	**2017**	0	1	0	1
**Total**		1	50	1	39

### Objective

The PLANES study (Placental Growth Factor Led Management of the Small for Gestational Age Fetus) has the following overall objectives: (1) to assess the feasibility of delivering sFlt-1/PlGF ratio led management of women with an SGA fetus, (2) to assess the acceptability of such an approach to women and clinicians, and (3) to explore the feasibility/acceptability of the study design.

Results from this feasibility study will inform the design of a future large randomised controlled trial (RCT) powered for adverse pregnancy outcome.

## Methods/design

The PLANES study is a randomised controlled feasibility study of standard care versus sFlt-1/PlGF ratio led management of pregnant women with an ultrasound diagnosis of an SGA fetus (defined as having an EFW < 10th percentile for gestation) at 32 + 0 to 37 + 6 weeks’ gestational age. The 10th centile will be defined by what is considered to be usual practice for each site; both sites in the pilot study use the customised GROW chart. This integrated mixed method study will also involve a health economic analysis and a perspectives work package exploring trial feasibility through interviews and questionnaires with randomised patients and their partners, as well as a clinician focus group and/or questionnaires and interviews.

### Participants

**Fig. 1:**
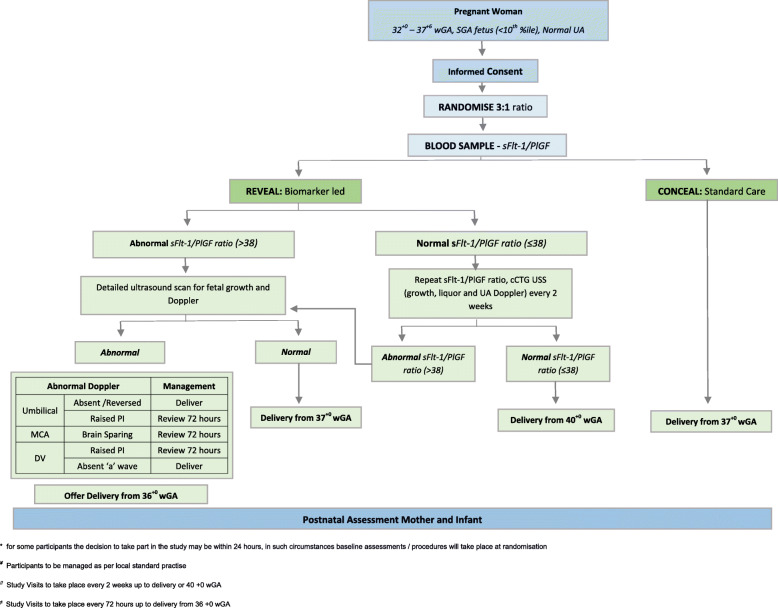
PLANES patient flow diagram

Inclusion criteria are women with a singleton pregnancy, confirmed SGA fetus (EFW < 10th centile on ultrasound within preceding 72 h), normal umbilical artery Doppler (pulsatility index < 95th centile), between 32 + 0 and 37 + 6 weeks of gestation, maternal age > 16 years old and able to give written informed consent. Exclusion criteria are known or suspected structural/chromosomal fetal abnormalities, either absent or reversed end diastolic flow in the umbilical artery on Doppler study, and severe maternal disease requiring urgent delivery. Participation in the perspectives work package follows the same criteria for participants, with the additional exclusion criteria of women who do not speak English. The flow of each participant from consent through to follow-up is shown in Fig. 1.

### Intervention

Following randomisation, women will be asked to provide a blood sample for assessment of sFlt-1/PlGF ratio; the result of which will be revealed (biomarker led) or concealed (standard care) from the attending clinical team.

Within the revealed/biomarker led care group, participants with a normal sFlt-1/PlGF ratio (≤ 38) will be advised that their risk of an adverse pregnancy outcome is low and will be offered delivery at 40 + 0 weeks gestation. Women will be offered further ultrasound and sFlt-1/PlGF ratios every 2 weeks, to ensure that they do not become high risk, with the care pathway adjusted if necessary (see Fig. 1). Participants with an abnormal sFlt-1/PlGF ratio (> 38) will be advised to attend for detailed ultrasound assessment by a fetal medicine expert within 72 h of the abnormal result being known. This assessment will involve fetal biometry and Doppler of the umbilical artery (UA), middle cerebral artery (MCA), and ductus venosus (DV). If Doppler studies are normal, then, delivery will be advised from 37 + 0 weeks [17]. If there is evidence of critical fetal compromise (absent end diastolic flow in the UA or absent a-wave in the DV), then, delivery will be performed as soon as feasible. If fetal Doppler studies are borderline (brain sparing (MCA pulsatility index < 5th centile) or increased resistance in UA or DV (pulsatility index > 95th centile)), the Doppler will be repeated every 72 h, and delivery will be offered between 36 + 0 and 37 + 0 weeks.

Women assigned to the concealed/standard care pathway will have an sFlt-1/PlGF ratio taken, but the result will be concealed from the clinical team with their pregnancy being managed as per the local NHS guideline with delivery from 37 + 0 weeks.

In all cases, women will receive cCTG on the same day as ultrasound assessment and a minimum of twice weekly. If at any point cCTG demonstrates a short-term variability (STV) < 3.0 ms, then, delivery should be planned [62]. If at any point the attending clinical team feels the need to deviate from a care pathway, they will also be able to do so, with outcomes recorded on an intention-to-treat basis. Women who prefer not to be randomised into the PLANES study will be offered the opportunity to give blood for a sFlt-1/PlGF ratio test with the result being concealed and not used to guide clinical management.

### Outcome measures

This study will assess the feasibility of using a blood biomarker, sFlt-1/PlGF ratio, to safely refine the care pathway for the management of women with an SGA fetus from 32 weeks of pregnancy. Post hoc analysis of sFlt-1 and PlGF individually will also be conducted. The outcomes for the study have been separated into those that determine the feasibility of a definitive trial and those that address whether the intervention might have an effect on neonatal outcome or healthcare costs. Our group has had previous successful experience employing this approach in the ReMIT-2 study [63].

The feasibility outcomes are as follows: number of eligible women at each site, the number of women recruited, the number of women randomised, the number of women not compliant with the intervention and the reasons for this, reasons for not participating (patients may still consent to the perspectives work package who do not wish to participate in the main study) and number of women lost to follow-up. Women’s and birth partner’s views on the approach to recruitment, including consent, decision-making and length and content of trial information materials and views on the sFlt-1/PlGF test will also be gathered in the perspectives work package. Clinicians’ views on the acceptability of a future trial including potential barriers to recruitment, consent decisions, trial procedures and clinician training needs via questionnaires and a focus group or interview will be collected.

The proof of concept outcomes for mother includes the following: gestation and frequency of induction of labour or planned caesarean, frequency of maternal hypertensive disorders, intensive care admission or maternal death prior to discharge. The proof of concept outcomes for the baby include stillbirth, neonatal death, Apgar score at 5 min < 7, umbilical artery pH < 7.05, birthweight < 10th centile, admission to neonatal unit and length of stay, use of therapeutic cooling, length of stay in hospital and duration of respiratory support.

### Sample size and recruitment

As a feasibility study, success will be determined on the acceptability of the management approach for women and clinicians. Participants will be recruited directly from the fetal medicine, antenatal clinic or maternity assessment units at the nominated research sites. It is proposed that the study should expect to recruit in the region of 100 participants across two sites over a 12 month period. This is a pragmatic figure which is based on the typical number of SGA patients seen per annum in large consultant-led NHS units within the UK.

Participants will remain in the study for a maximum of 8 weeks (from 32 + 0 weeks [earliest point of eligibility] to estimated due date (EDD)). The study will end when the last recruited woman/baby is discharged from hospital after birth (up to age 1 month uncorrected) or the baby has reached their EDD.

### Enrolment and consent

Prior to taking part in the study, all women will have confirmation of their SGA status completed by their attending clinician based on an ultrasound scan performed within the preceding 72 h. Once a potential participant has been identified (all eligibility criteria met), they will be invited to take part in the study and a member of the clinical research team at site will discuss this with them. At this point, the woman and partner will receive written (PLANES patient information sheet (PIS)) and verbal information on the PLANES study, as well as an opportunity to ask questions and take any additional time required to consider taking part in the study. All potential participants will be given a unique screening ID that will subsequently be used to detail the reasons for the continuation or discontinuation at the screening stage. Participants willing to proceed will be asked to sign the study-specific informed consent form (ICF), and once written informed consent has been provided, participants will be registered onto the study and randomised by the research midwife/clinician at site.

The PLANES PIS will also include information regarding taking part in the perspective work package. If women are happy to take part in this, they will be asked to complete the relevant sections of the study ICF. Women who decline to take part in the main study will also be asked if they would like to take part in this perspective work package, and these participants will be expected to complete the standard participant ICF, initialling only those boxes relevant to the perspective study.

The research team are conscious that women may not feel that they should deviate from ‘normal’ NHS care despite the more detailed assessments of fetal wellbeing PLANES have set in place. In order to increase the ability of this study to inform a future RCT powered to prevent stillbirth, we will ask women who decline to be randomised whether they would consent to a single sFlt-1/PlGF ratio being taken and stored for processing only after the study has ended. In this way, we may still gain valuable information about the ability of this test to predict clinically relevant pregnancy outcomes even if women do not wish to be randomised.

The critical data in the PLANES study is derived from blood samples. Refusal to give the crucial blood sample at randomisation would result in significant compromise to the study. Therefore, any participant who does not provide this sample would need to be withdrawn from the study. Participants can refuse any further subsequent blood sample and remain in the study under the intention-to-treat principle.

### Randomisation

As there is greater value in the outcomes and opinions of those women undergoing the intervention, participants will be randomised to receive revealed (biomarker led) or concealed pathways in a ratio of 3:1. Patients will be randomised by authorised site staff using an electronic randomisation system, accessed by delegated site staff using a secured password-protected website. The randomisation code list will be generated on the basis of randomly permuted blocks by a Liverpool Clinical Trials Unit (LCTU) statistician using the ‘ralloc’ command with the software package STATA. LCTU information security staff at the University of Liverpool will be responsible for designing and supporting the PLANES randomisation programme. It is not possible to blind the participant, or their attending midwife or clinician, to their allocated pathway at randomisation as the sFlt-1/PIGF ratio taken in the biomarker-led pathway will inform further management.

### Trial assessments and procedures

A prerequisite prior to randomisation of the participant into the PLANES study is a review of the participants medical and medication history; fetal assessments including computerised CTG and fetal ultrasound; and maternal observations including blood pressure, pulse and urinalysis. Once informed consent has been obtained, the participant will be asked to provide a blood sample for assessment of the sFlt-1/PlGF ratio. The participant will then be randomised to either the revealed or concealed pathway. At this time, the patient will also complete an EQ-5D-5L health questionnaire, and those consented to the perspectives work package will undertake a questionnaire and with additional patient consent either face-to-face or telephone interviews.

Continuing assessments will vary depending on the participant’s care pathway within the study; those in the control/concealed pathway receiving standard care and assessments on adverse events and standard care, outcomes will take place at the end of study. Participants within the revealed/biomarker led pathway will undergo blood pressure, pulse, urinalysis, computerised CTG and fetal ultrasound assessment as well as repeat sampling for sFlt-1/PlGF ratio as detailed below dependent on previous sFlt-1/PlGF ratio results and Doppler studies. In all cases, women will receive cCTG on the same day as ultrasound assessment and a minimum of twice weekly. Further assessments will take place after delivery and before discharge from hospital, and this will include a repeat EQ-5D-5L health questionnaire, childbirth experience questionnaire and recording of delivery outcomes, and following this in the postnatal period, assessments on maternal and neonatal outcomes will be completed (See Table 2).

**Table 2:** Schedule of study-related assessments/procedures

Procedure		^a^Screening	^a^Baseline	Study visit 1	Study visit 2	Study visit 3	Delivery	Postnatal	End of study
**Global outcomes**									
Review of medical history		**X**							
Review of medication		**X**							
Maternal assessment	Blood pressure	**X**		**X**	**X**	**X**			
	Pulse	**X**		**X**	**X**	**X**			
	Urine dip	**X**		**X**	**X**	**X**			
Fetal assessment	Ultrasound (growth, liquor, and UA Doppler)	**X**							
	cCTG	**X**							
Eligibility assessment		**X**							
Informed consent			**X**						
Randomisation			**X**						
Blood sample collection			**X**	**X**	**X**	**X**			
Qualitative: women/partner			**X**						**X**
Qualitative: clinician									**X**
Health economic: EQ-5D-5 L			**X**				**X**		
Health economic: CEQ							**X**		
Delivery outcomes							**X**		
Maternal postnatal outcomes								**X**	
Neonatal postnatal outcomes								**X**	
End of study outcomes									**X**
^**b**^**Concealed—standard care pathway**									
Adverse event reporting									**X**
Collection of standard care outcomes									**X**
^**c**^**Revealed—normal group pathway**									
Maternal assessment	BP			**X**	**X**	**X**	**X**	**X**	
	Pulse			**X**	**X**	**X**	**X**	**X**	
	Urine dip			**X**	**X**	**X**			
Fetal assessment	Ultrasound (growth, liquor, and UA Doppler)			**X**	**X**	**X**			
	cCTG			**X**	**X**	**X**			
Adverse event reporting				**X**	**X**	**X**	**X**	**X**	**X**
Blood sample collection				**X**	**X**	**X**			
^**d**^**Revealed—abnormal group pathway**									
Maternal assessment	BP			**X**	**X**	**X**	**X**	**X**	
	Pulse			**X**	**X**	**X**	**X**	**X**	
	Urine dip			**X**	**X**	**X**			
Fetal assessment	Ultrasound (growth, liquor and UA Doppler)			**X**	**X**	**X**			
	cCTG			**X**	**X**	**X**			
	MCA and DV Doppler			**X**	**X**	**X**			
Adverse event reporting				**X**	**X**	**X**	**X**	**X**	**X**

^a^For some participants, the decision to take part in the study may be within 24 h; in such circumstances, baseline assessments/procedures will take place at randomisation

^b^Participants to be managed as per local standard practise

^c^Study visits to take place every 2 weeks up to delivery or 40 + 0 wGA

^d^Study visits to take place every 72 h up to delivery from 36 + 0 wGA

Clinicians at site at the end of the study will be asked to participate in focus groups, and for those who are unable to attend, online questionnaires and interviews will also be undertaken.

### Adverse event reporting

All adverse events for this study will be recorded at each study visit, the condition of each participant monitored throughout the study until 1 month postnatal. The intervention to which participants are randomised to as part of this study provides additional care to that which is usually provided as part of local standard care and therefore large numbers of serious adverse events are not anticipated. SAE to be reported are that of intrauterine fetal death (stillbirth), maternal death and neonatal death; all of which are also pre-specified outcomes for the study. Less serious adverse events (preterm delivery, pre-eclampsia, caesarean section, admission to neonatal unit) are exempt from immediate safety reporting due to the anticipation of these in SGA pregnancies—unless a causal relationship to the study design is suspected.

### sFlt-1/PlGF ratio blood tests

The sFlt-1/PlGF ratio will be used to guide intervention in the study; Roche® has agreed to provide the sFlt-1/PlGF ratio kits for no cost. Blood samples relating to the intervention pathway collected at Liverpool Women’s Hospital and St Mary’s Hospital will be analysed within the Liverpool Clinical Laboratories at the Royal Liverpool and Broadgreen University Hospital NHS Trust and the Manchester University NHS Foundation Trust, respectively. Additional samples that will be collected from both sites will be sent to the Centre for Women’s Health Research laboratories where they will be processed and stored until the end of the study.

In order to get the most information possible during the study period, we will be asking women to allow us to use the remaining blood taken during PLANES to be used in other ethically approved research, a process called ‘gifting’. Since the initial application of the PLANES study, other companies have begun to produce PlGF biomarker tests (Perkin-Elmer and Quidel), and we will perform post hoc analysis of blood samples with these companies to ascertain whether they could be used in the future management of SGA. Neither of these tests will however be used to determine clinical care pathway during the study period.

### Data management

Study data will be captured using electronic case report forms (eCRFs) transcribed to a bespoke study database with participants identified only by their unique participant identification number allocated at randomisation. It will be accessed via a secure webpage by delegated research site staff and is designed and maintained by the LCTU. All eCRF data entered into the study database will be centrally monitored by the Centre for Women’s Health Research to ensure that data collected is consistent with adherence to the study protocol. The database also includes validation features which will alert the user to certain inconsistent or missing data on data entry, and if any problems are identified via automated validation or central monitoring, a query is raised and emailed to site. Regular reports will be generated to identify discrepancies in the data and allow for follow-up. Electronic and paper screening logs will also be kept in clinics to record the number of patients declining participation and when volunteered the reason given; all of which will be kept in a secure locked location on NHS premises.

### Statistical analysis

Analysis of study data will take place once all participants have received the planned follow-up and all data is available. The likelihood of missing data is small given the standard procedure in place to manage the study centrally. Therefore, final analyses will take place on a complete-case basis with no adjustments made (e.g. multiple imputation) in the case of missing data.

A statistical analysis plan will be determined and finalised prior to final data lock. As the analyses being carried out are based on feasibility, the details in terms of the methodology may be altered during the course of the study. Patients will be summarised on an intention-to-treat basis retaining all patients irrespective of any protocol deviations. Further secondary analysis will be carried out on a per protocol population. Further analyses may be carried out on planned subgroups (e.g. those who meet the inclusion criteria for a future study) as is required. Multivariate data analysis techniques will be also used to attempt to find natural groupings in the generated data including hierarchical cluster analysis and principle component analysis. As this is an exploratory study, no formal levels of significance are set. All statistics presented will be presented alongside 95% confidence intervals so as to give an indication of the level of precision only. Continuous data will be summarised as median, interquartile range (IQR) and ranges, and categorical data shall be summarised as frequencies of counts and associated percentages. Quantitative analysis will involve simple descriptive statistics and the chi-square test for trend. Data from each method will be analysed separately then synthesised through the use of constant comparative analysis.

### Perspective work package

The perspective work package will include clinicians involved in PLANES as well as women, and their partners, who have provided consent to be contacted during enrolment into the PLANES study. Questionnaires and interviews will involve women in both the concealed and revealed pathways as well as those who declined randomisation. This will allow for meaningful exploration of different experiences on the approach to recruitment in the PLANES study, consent, decision-making and length and content of trial information materials. Each patient and their partner (if applicable) will be provided with the PLANES questionnaire. The PLANES researcher will make contact with women and their partners (if applicable) to arrange an interview within approximately 1 month of consent. All interviews will be conducted using the PLANES women and partner interview topic guide which has been informed by previous pilot trials within the NHS, and respondent validation will be used so previously unanticipated topics can be added as analyses progress [64, 65]. The University of Liverpool PLANES Qualitative study team will conduct all focus groups and interviews. Interviews will be conducted until data saturation point; this is anticipated to be 15–25 interviews and approximately 50 questionnaires (48% response rate).

Clinicians involved in the PLANES study will be sent an email invitation to participate in a focus group at the end of the PLANES study recruitment period. The focus group (approximately 8–10 participants) will incorporate the use of voting software so that both qualitative and quantitative data are collected. All focus groups and interviews will be conducted using the clinician focus group and interview topic guides which will be informed by interim findings from women and birth partner questionnaires and interviews. Those unable to attend the focus group will be invited to participate in an interview and an online questionnaire.

Thematic analysis of qualitative data from the interviews, questionnaires, and focus groups will be assisted using NVivo 10 qualitative data analysis package and the SPSS software for statistical analysis. Whilst data will be analysed thematically, the focus will be modified to fit with the criterion of catalytic validity, whereby findings should be relevant to future research and practice (in particular, insight into trial acceptability and the design of the potential definitive RCT) [66, 67].

### Health economic analyses

By performing sFlt-1/PlGF ratio on women with SGA babies, we hope to be able to reduce the likelihood of intervention in those with a normal result, in turn reducing the number of preterm and early term deliveries and associated neonatal care. In those with abnormal results, greater recognition of clinical concern and more detailed assessments may improve the outcomes for these high-risk pregnancies. All of these outcomes have important cost implications that would need further, comprehensive assessment in a larger definitive trial.

The overarching aim of the economic analysis embedded in this study is to assess the feasibility of collecting relevant information on key economic outcomes. Such outcomes include health care resource use (e.g. care related to birth and complications, cost of additional diagnostic tests) and relevant structured quantitative outcomes, related to childbirth experience and maternal health-related quality of life. Childbirth experience will be captured through the use of the Childbirth Experience Questionnaire administered shortly after delivery [68, 69]. Quality of life will be collected through the widely used EuroQol 5D-5 L instrument, which will be administered before and after delivery [70]. The quality and completeness of the collected data will be assessed, and findings will inform data collection methods and schedules in the subsequent RCT.

### Trial governance

The PLANES study will have a Trial Management Group (TMG), Trial Steering Committee (TSC) and an Independent Safety and Data Monitoring Committee (ISDMC) to monitor the study progress.

The TMG will be responsible for the day-to-day running and management of the study and will be comprised of the CI and other lead investigators/core study management staff. The TSC will provide oversight of the study, concentrating on progress of the study, adherence to protocol, participant’s safety and consideration of new information, making recommendations on study pathway modifications and continuation of the study.

The ISDMC will be responsible for reviewing and assessing recruitment, interim monitoring of safety and effectiveness, trial conduct and external data. A sub-committee will also meet to provide ongoing review on any maternal, fetal or neonatal deaths reported on the study as SAEs and to provide ongoing review of AE’s. The ISDMC will also provide recommendations to the TSC concerning continuation of the study.

## Discussion

PLANES will assess the ability to modify the care of women carrying a SGA fetus at late preterm and term gestations using the placental biomarker sFlt-1/PlGF ratio with the aim of safely delaying delivery to a later gestation than currently advocated. The PLANES study will provide data on the acceptability of this management to participants and clinicians as well as the cost implications of the proposed intervention; both of which are important in determining if a larger RCT is feasible. The results from this study will be used to inform a future large randomised controlled trial investigating the effectiveness and cost-effectiveness of sFlt-1/PlGF led management of SGA fetuses, powered for adverse pregnancy outcome.

It is evident that more research is needed into the optimal management and timing of delivery of an SGA fetus. The current blanket approach by the RCOG [17] and many other national guidelines may reduce the risk of term stillbirth; however, this comes at the detriment of a large proportion of SGA pregnancies undergoing early term deliveries who may not have needed this intervention and may increase the long-term risks from earlier delivery which could have been avoided. Biomarkers for placental function may hold the key to identifying those pregnancies truly at risk of adverse outcomes, allowing for individualised care and reduction in intervention; however, at present, there is insufficient evidence to make a judgement regarding the efficacy of this approach [71]. Initial studies of the implementation of sFlt-1/PlGF ratio suggest that using biomarkers to determine requirement for or the timing of intervention is feasible [72]. More research and intervention trials in this area will not only address the priorities identified in the recent Saving Babies’ Lives Care Bundle but will help further develop guidance on the management of the SGA fetus [36].

## Trial status

Trial registration: ISRCTN58254381. Protocol Version 2.0 7 August 2019. Recruitment due to commence June 2020

## Data Availability

Data sharing is not applicable to this article as no datasets were generated or analysed during the current study.

## Abbreviations

AE: Adverse event
AGA: Appropriate for gestational age
CI: Chief investigator
CfWHR: Centre for Women’s Health Research
eCRF: Electronic case report form
CTG: Cardiotocography
cCTG: Computerised cardiotocography
CTU: Clinical trials unit
EDD: Estimated delivery date
EDF: End diastolic flow
EFW: Estimated fetal weight
GROW: Gestation-related optimal weight
HCA: Hierarchical cluster analysis
HRA: Health Research Authority
GCP: Good Clinical Practice
FGR: Fetal growth restriction
ICH: International Conference on Harmonisation
ICF: Informed consent form
IOL: Induction of labour
IQR: Interquartile range
IS: Information security
ISDMC: Independent safety and data monitoring committee
LCTU: Liverpool Clinical Trials Unit
LWH: Liverpool Women’s Hospital
MCA: Middle cerebral artery
NICE: National Institute for Health and Care Excellence
NICU: Neonatal intensive care unit
NIHR: National Institute for Health Research
PI: Principal investigator
PID: Participant identification number
PIS: Participant information sheet
PlGF: Placental growth factor
RCOG: Royal College of Obstetricians and Gynaecologists
RCT: Randomised controlled trial
REC: National Research Ethics Committee
RfPB: Research for Patient Benefit
SAP: Statistical analysis plan
SFH: Symphysial fundal height
s-Flt1: Soluble fms-like tyrosine kinase-1
SGA: Small for gestational age
SAE: Serious adverse event
STV: Short-term variability
TSC: Trial Steering Committee
TMG: Trial Management Group
UA: Umbilical artery
wGA: Weeks’ gestational age
WP: Work package
